# Supplementary material for: Depletion of Alpha-Melanocyte-Stimulating Hormone Induces Insatiable Appetite and Gains in Energy Reserves and Body Weight in Zebrafish
Source: Biomedicines. 2021 Aug 2;9(8):941. doi: 10.3390/biomedicines9080941 (PMC8392443; doi:10.3390/biomedicines9080941)
Supplement: Supplementary file 1 [file biomedicines-09-00941-s001.zip › biomedicines-1296783-supplementary.pdf]

Supplementary Table S1. Primer sequences used for quantitative RT-PCR

Gene	Accession	Forward Primer	Reverse Primer
bactin1	NM_131031.1	CGAGCAGGAGATGGGAACC	CAACGGAAACGCTCATTGC
gh1	NM_001020492	TCGTTCTGCAACTCTGACTCC	CCGATGGTCAGGCTGTTTGA
ghrb	NM_001111081.1	GCTGCGCTCTGTTGATAATGT	GGCGGAGGGAGGTGGAT
igf2b	NM_001001815	AACCTGCCAAGTCAGAGAGGG	GGACCTCCTGTTTTAATGCGG
igfbp1b	NM_001098257.2	GTCACAACTCAACGCCATCC	GAACTTCTCTCCCAACGCCT
igfbp5b	NM_001126463.1	CAGGGGAGTGTGTACGAACG	AGTCGTGTCTGGCTTCACTG
igfbp6b	NM_001161402.2	CATCGCTTCTCCTACGGGAC	GGGGTCTCGAACTCACCATT
pmch	NM_001202542.1	TTCATACTTGCGGACACAGG	TCCATCGTGCTGAATCCATC
agrp2	NM_001271291.1	AGTTCAGTCCATCACATGCG	TTTCTCCTGATTCCACACTCC
npy	NM_131074.2	AGCACTAAGACACTACATCAACC	GATGAGATCACCATGCCAAATG
hcrt	NM_001077392	TCATGGACTGCACAGCTAAG	CATCTCGTAGAGTTTGCAGGAG
bdnf	NM_001308648.1	AGCATCTGTTGGAGTGTGTG	CAGCTCTCATGCAACTGAAG
sim1a	NM_178222.3	CTGTACATTTCAGAAACGGCG	AAGTGTGAATGATAGGGCTGG
crhb	NM_001007379.1	CCAAGGATTTACCAATTACGCAC	CGATGGCTCTACATTCATACGG
trh	NM_001012365.2	GCGCTCCATCCTCACAC	GCTGTCGCTTCTCCATCC
pcsk2	NM_001142266	TCTCGCCTCAAACTACAACG	TGGAAACGGCAGACACTTC
ghrl	NM_001083872.1	GTGTTTCTCTTTCCTTGTGTCTC	TTCTTTGATCACTGGTATCTCTGG

Supplementary Table S2. Primer sequences used for in situ hybridization probes

Gene	Accession	Forward Primer	Reverse Primer
pomca	NM_181438	ACAGAGGAGAACATCTTGGAATGC	GCCCACCTTCGTTTCTATGCATGA
bdnf	NM_001308648.1	GTCATTGAGGAGTTGCTTGAG	TATCTGCCCCTCTTA ATGGTC
gh1	NM_001020492	ATCCGTGTGCAACACCTTCACCAG	CCATGCCTGCTTGATATTTCCTGC
sim1a	NM_178222.3	TGTTGCTGGTAAAAGGACAAG	CCCATTCTCAGGTGAAGATTT
